# AlzhCPI: A knowledge base for predicting chemical-protein interactions towards Alzheimer’s disease

**DOI:** 10.1371/journal.pone.0178347

**Published:** 2017-05-25

**Authors:** Jiansong Fang, Ling Wang, Yecheng Li, Wenwen Lian, Xiaocong Pang, Hong Wang, Dongsheng Yuan, Qi Wang, Ai-Lin Liu, Guan-Hua Du

**Affiliations:** 1 Institute of Clinical Pharmacology, Guangzhou University of Chinese Medicine, Guangzhou, China; 2 Department of Encephalopathy, The Second Affiliated Hospital of Guangzhou University of Chinese Medicine, Guangzhou, China; 3 Guangdong Provincial Key Laboratory of Fermentation and Enzyme Engineering, Pre-Incubator for Innovative Drugs & Medicine, School of Bioscience and Bioengineering, South China University of Technology, Guangzhou, China; 4 Institute of Materia Medica, Chinese Academy of Medical Sciences and Peking Union Medical College, Beijing, PR China; National Chiao Tung University College of Biological Science and Technology, TAIWAN

## Abstract

Alzheimer's disease (AD) is a complicated progressive neurodegeneration disorder. To confront AD, scientists are searching for multi-target-directed ligands (MTDLs) to delay disease progression. The *in silico* prediction of chemical-protein interactions (CPI) can accelerate target identification and drug discovery. Previously, we developed 100 binary classifiers to predict the CPI for 25 key targets against AD using the multi-target quantitative structure-activity relationship (mt-QSAR) method. In this investigation, we aimed to apply the mt-QSAR method to enlarge the model library to predict CPI towards AD. Another 104 binary classifiers were further constructed to predict the CPI for 26 preclinical AD targets based on the naive Bayesian (NB) and recursive partitioning (RP) algorithms. The internal 5-fold cross-validation and external test set validation were applied to evaluate the performance of the training sets and test set, respectively. The area under the receiver operating characteristic curve (ROC) for the test sets ranged from 0.629 to 1.0, with an average of 0.903. In addition, we developed a web server named AlzhCPI to integrate the comprehensive information of approximately 204 binary classifiers, which has potential applications in network pharmacology and drug repositioning. AlzhCPI is available online at http://rcidm.org/AlzhCPI/index.html. To illustrate the applicability of AlzhCPI, the developed system was employed for the systems pharmacology-based investigation of shichangpu against AD to enhance the understanding of the mechanisms of action of shichangpu from a holistic perspective.

## Introduction

Alzheimer’s disease (AD) is the most common neurodegenerative disease in elderly people, which is accompanied by the progressive impairment of memory and cognitive function [1]. The pathological hallmarks of AD are mainly characterized by extracellular senile plaques (SPs) and intracellular neurofibrillary tangles (NFTs), as well as selective cholinergic neuronal loss [2]. Current drugs for AD treatment that target cholinergic and glutamatergic neurotransmission, such as donepezil and memantine, show limited benefits to most AD patients [3, 4]. Therefore, there is an urgent need to develop an effective treatment that could not only improve symptoms but also modify the disease process.

The aetiology of AD is multifactorial. Considering the complexity of AD, the classic “one drug, one target” solution is not effective enough [5]. Indeed, many research projects in the field have been focused on developing multi-target/multifunctional therapies to modify the disease process [6–9]. Experimental identification of hits that interact with multiple proteins is costly, time consuming, and labour intensive. *In silico* target prediction is a fast and cheap alternative to experimental target identification approaches, which could accelerate the discovery of “multi-target-directed ligands (MTDLs)” against AD.

The central issue of target prediction is to identify the chemical-protein interactions (CPI) between chemicals and proteins. Two main computational methods are used to predict the CPI for a given ligand, which were summarized by a recent review [10]. The methods are the ligand-based target prediction (LBTP) approach [11, 12] and the structure-based target prediction (SBTP) approach [13, 14]. As an LPTP approach, the multi-target quantitative structure-activity relationship (mt-QSAR) method is highly predictive and convenient and can simultaneously predict activities against different targets by using large and heterogeneous chemical datasets [15]. Cheng *et al*. built 200 mt-QSAR models for 100 GPCRs and 100 kinases using the support vector machine (SVM) algorithm and found that the models performed better than that built using the chemogenomic method [16].

Inspired by Cheng’s work [16], we built 100 binary classifiers to predict the chemical-protein interactions for 25 key targets against AD using the mt-QSAR method. The validated models were used to explore the polypharmacology against AD, and the prediction results were confirmed by the reported bioactivity data and our *in vitro* experimental validation, resulting in several highly potent MTDLs [17]. However, there are still some pitfalls and disadvantages that limit their application. First, the models only include drug candidate targets that entered into phase I clinical trials, excluding those in preclinical trials. Second, it is inconvenient and unscientific that no criteria for target naming and classification are defined. Furthermore, no publicly available knowledge base has been developed to integrate the binary classifiers that we built. Thus, it is still necessary to improve and update this research to predict CPI towards AD.

**Fig 1:**
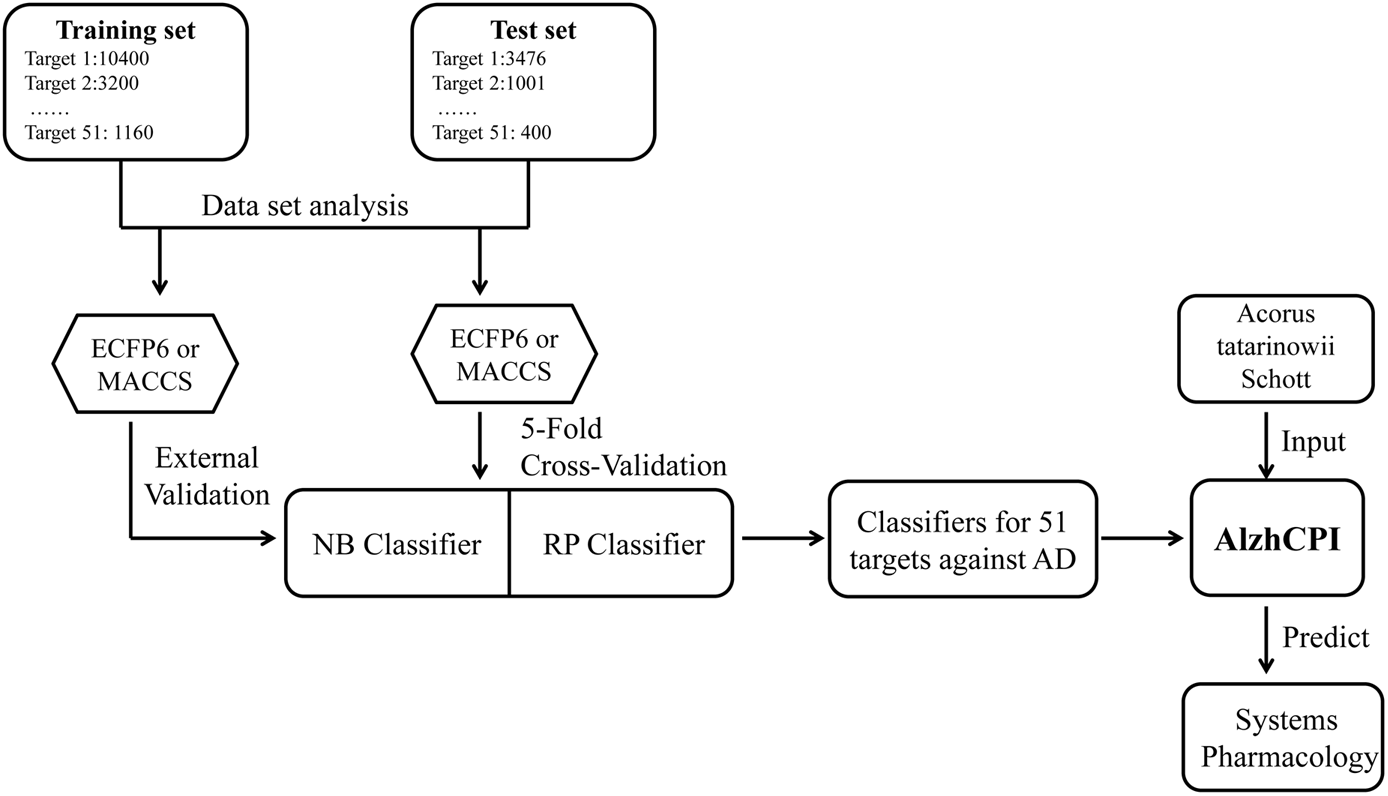
The schematic workflow of AlzhCPI to predict cheimical-protein interactions toward Alzheimer's disease based on the multitarget quantitative structure-activity relationships (mt-QSAR).

The current work aims to apply the mt-QSAR method to enlarge the model system (AlzhCPI) to predict CPI towards AD. The schematic workflow of AlzhCPI is shown in Fig 1. Based on the naive Bayesian (NB) and recursive partitioning (RP) algorithms, the updated system assembled 204 binary classifiers to integrate the chemical and pharmacological information derived from the BindingDB database. All developed classifiers were validated by 5-fold cross-validation and test set validation. To provide a free service for the scientific community, a web server named AlzhCPI was developed to integrate comprehensive information approximately 204 binary classifiers into a web-based information system. To illustrate examples of AlzhCPI, the developed system was employed for systems pharmacology-based investigation of shichangpu against AD, which aided in analysing the mechanisms of action of shichangpu.

## Materials and methods

### Data set construction

**Fig 2:**
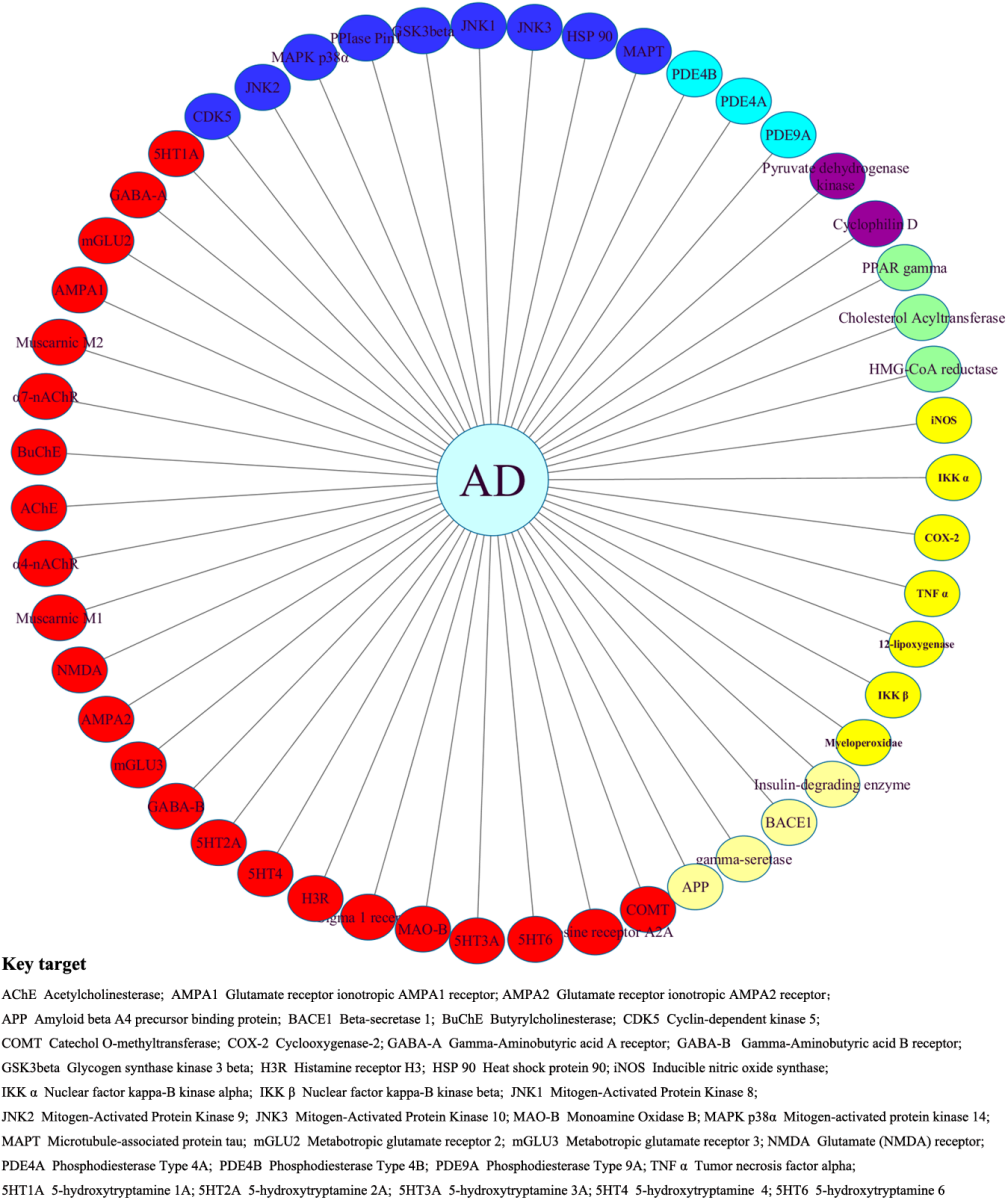
Summary of 51 key targets in AlzhCPI.

Following a similar procedure to the previous study, the Thomson Reuters Integrity Database [18], the Therapeutic Target Database (TTD) [19], and text mining from references [20–22] were used to collect targets for AD in preclinical trials, resulting in 26 preclinical targets. Together with 25 important targets that had entered into at least phase I clinical trials, 51 targets related to AD were obtained (Fig 2). After that, the names of the targets were imported into the UniProt database [23] to acquire the corresponding encoding gene, UniProt ID, entry name, and standardized protein name (S1 Table). The chemical structures and bioactivity data of the ligands for the 26 preclinical targets were downloaded from the Binding Database (http://www.bindingdb.org, accessed July 2015) [24].

The ligands were standardized using the following criteria: (i) duplicate molecules were deleted; (ii) salts were converted to the corresponding acid or base and solvent molecules were removed from hydrates; and (iii) the molecule was considered to be positive (designated +1) if its Ki, EC_50_ or IC_50_ ≤ 10 μM. After filtering, 21,468 active ligands were got. The decoy compounds (designated -1) for 26 targets were mainly generated through three ways (S2 Table): (i) randomly extracted from the specs database; (ii) directly extracted from DUD subsets; and (iii) generated in the DUD online database with known active compounds. The ratio of decoys to active ligands is 3. Both the active and decoy compounds were randomly divided into two groups (training set and test set at a ratio of 3).

### Chemical descriptors calculation

Two kinds of fingerprints were calculated for the description of the small molecules. The first was the ECFP_6 fingerprint, which was calculated by the Discovery Studio 4.0 software [25]. Extended connectivity fingerprints (ECFP) represents a much larger set of features than a set of predefined substructures. The other was the MACCS fingerprint computed by PaDEL-Descriptor 2.18 [26]. MACCS used a dictionary of MDL Public Keys, which contains the 166 most common substructure patterns. A detailed description of these fingerprints can be found in the original literature [27, 28].

### mt-QSAR method

In traditional QSAR studies, one binary classifier can only predict the activity of a compound against one specific target. The essence of mt-QSAR is to decompose the multi-label problem into multiple binary classification problems. As a consequence, to predict one molecule against 26 preclinical targets related to AD, 104 mt-QSAR classifiers were constructed based on two fingerprints (ECFP_6 and MACCS) and two machine learning algorithms (naive Bayesian and recursive partitioning). For each target, four classifiers (NB_ECFP6, NB_MACCS, RP_ECFP6 and RP_MACCS) can be used to predict the activity of a given molecule.

#### Naive Bayesian

The naive Bayesian (NB) models were developed using Discovery Studio 4.1 [25]. An advantage of NB classifiers is that they can process an abundance of data, can learn fast and are tolerant of random noise. A more detailed introduction can be found in the following references [29, 30]. In general, NB is a simple probabilistic classifier based on applying Bayesian theory with strong (naive) independence assumptions, which relates the conditional and marginal probabilities of two events. It generates the posterior probabilities based on the core of the function, given by Eq 1. The specific meaning of each parameter can be found in our previous study.

$$P\left(+|A_{1},\ldots ,A_{n}\right)=\frac{P\left(A_{1},\ldots ,A_{n}|+\right)P\left(+\right)}{P\left(A_{1},\ldots ,A_{n}\right)}$$ (1)

#### Recursive partition

Recursive partitioning (RP), using Discovery Studio 4.1 [25], was applied to develop decision trees to categorize the data set into active compounds and decoys. RP is a statistical method for multivariable analysis that operates by developing a decision tree to classify the members. Models are constructed by successively splitting a data set into smaller and smaller subsets using a set of hierarchical rules. The result of an RP model is more intuitive than other algorithms because it can be demonstrated by a “decision tree” or “graph” [31, 32].

In this study, 5-fold cross-validation was adopted to determine the degree of pruning to obtain the best predictive accuracy. The specific parameters were set as follows: minimum number of samples at each node and maximum tree depth, where the maximum tree depth was 10, 20 and 20.

### Measurement of prediction quality

The internal 5-fold cross-validation and external test set validation were applied to evaluate the training sets and test set, respectively. In a 5-fold cross-validation, the entire data set was equally divided into 80% samples for training the model and 20% data samples for an internal validation set.

The quality of all Bayesian and RP classifiers was evaluated based on the quantity of true positives (TP), true negatives (TN), false positives (FP) and false negatives (FN). The sensitivity (SE), specificity (SP), overall prediction accuracy (Q), and Matthews correlation coefficient (MCC) were further calculated using Eqs 2–5, respectively.

$$\text{SE}=\frac{\text{TP}}{\text{TP}+\text{FN}}$$ (2)

$$\text{P}=\frac{\text{TN}}{\text{TN}+\text{FP}}$$ (3)

$$\text{Q}=\frac{\text{TP}+\text{TN}}{\text{TP}+\text{TN}+\text{FP}+\text{FN}}$$ (4)

$$\text{MCC}=\frac{\text{TP}\times \text{TN}-\text{FN}\times \text{FP}}{\sqrt{\left(\text{TP}+\text{FN}\right)\left(\text{TP}+\text{FP}\right)\left(\text{TN}+\text{FN}\right)\left(\text{TN}+\text{FP}\right)}}$$ (5)

In addition, the area under the receiver operating characteristic (ROC) curve (AUC) was also calculated. The ROC curve shows the separation ability of a binary classifier by iteratively setting the possible classifier threshold [33]. The AUC value falls in the range of 0.5≤AUC≤1. AUC = 1.0 means a perfect classifier, whereas AUC = 0.5 indicates the classifier has no discriminative power.

### Compound filtering in the case study

A total of 132 chemical structures in the herb *Acorus tatarinowii Schott* (shichangpu) were obtained from the Traditional Chinese Medicine System Pharmacology Database [34] (TCMSP, http://tcmspnw.com), the potential target database of TCM [35] (TCM-PTD, http://tcm.zju.edu.cn/ptd), the Traditional Chinese Medicine Integrated Database [36] (TCMID, http://www.megabionet.org/tcmid/) and relevant references [37, 38]. Given that the content of most chemicals was very low, 22 typical ingredients with contents in the volatile oil higher than 0.1% were kept for further study, according to previous publications [39, 40]. The SMILES structure of the 22 compounds are given in S3 Table.

### Target prediction for approved drugs and shichangpu against AD

The putative targets for approved drugs and shichangpu against AD were predicted by AlzhCPI. Considering that each classifier has its strengths and weaknesses, it is more reasonable to predict the activity of one given compound by combining the results from the four classifiers. Herein, a chemical-protein interaction is defined as a potential interaction if the molecule is predicted to be active by at least two out of the four single classifiers within one target.

### Network construction and analysis

To reveal the underlying mode of action between compounds and targets, compound-target networks were constructed. The networks were generated and analysed using Cytoscape 3.2.0 [41]. The degree of a node was calculated by the network analysis plugin in Cytoscape, which defines the number of edges connected to a node, implying the significance of the node in a network.

## Results and discussion

### Data set analysis

To explore the chemical diversity of the data set used in the training set and test set, the Tanimoto similarity index was calculated using the ECFP_2 fingerprint in Discovery Studio 4.1 [25]. Tanimoto similarity index is an indicator to reflect chemical diversity within a data set, and a smaller value indicates that compounds within the data set have better diversity. As given in Table 1, similar to previous results for 25 targets, the Tanimoto indexes range from 0.054 to 0.338 for 26 training sets and 0.013 to 0.270 for 26 test sets, which indicates that the entire data set of 51 targets is diverse enough.

**Table 1:** Detailed statistical description of the entire data set based on the multi-label classification strategy.

Encoding Gene	Training set (ECFP2)				Test set (ECFP2)			
	Inhibitors	decoys	Total	Tanimoto index	Inhibitors	decoys	Total	Tanimoto index
HTR2A	2200	6600	8800	0.288	742	2226	2968	0.198
ADORA2A	2360	7080	9440	0.279	783	2349	3132	0.179
CHRM2	380	1140	1520	0.249	128	384	512	0.15
PDE9A	110	330	440	0.114	33	99	132	0.046
GRM2	310	930	1240	0.28	106	318	424	0.234
GRM3	50	150	200	0.305	16	48	64	0.203
MAPK8	780	2340	3120	0.192	266	798	1064	0.091
MAPK9	330	990	1320	0.13	108	324	432	0.06
MAPK10	510	1530	2040	0.183	174	522	696	0.056
MAPK14	40	120	160	0.181	19	57	76	0.171
HS90AA1	750	2250	3000	0.215	248	744	992	0.1361
PIN1	60	180	240	0.125	23	69	92	0.0544
MAPT	40	120	160	0.1125	12	36	48	0.0209
PTGS2	1760	5280	7040	0.542	583	1749	2332	0.164
NOS2	570	1710	2280	0.33	184	552	736	0.288
MPO	60	180	240	0.338	19	57	76	0.211
CHUK	120	360	480	0.173	41	123	164	0.098
IKBKB	600	1800	2400	0.22	198	594	792	0.123
TNF	560	1680	2240	0.184	192	576	768	0.083
ALOX12	120	360	480	0.2	40	120	160	0.119
CTSD	1250	3750	5000	0.246	423	1269	1692	0.093
PDK1	440	1320	1760	0.261	149	447	596	0.2
HMGCR	600	1800	2400	0.233	199	597	796	0.136
IDE	60	180	240	0.054	20	60	80	0.013
PPARG	1730	5190	6920	0.264	582	1746	2328	0.171
CES1	290	870	1160	0.305	100	300	400	0.27

**Fig 3:**
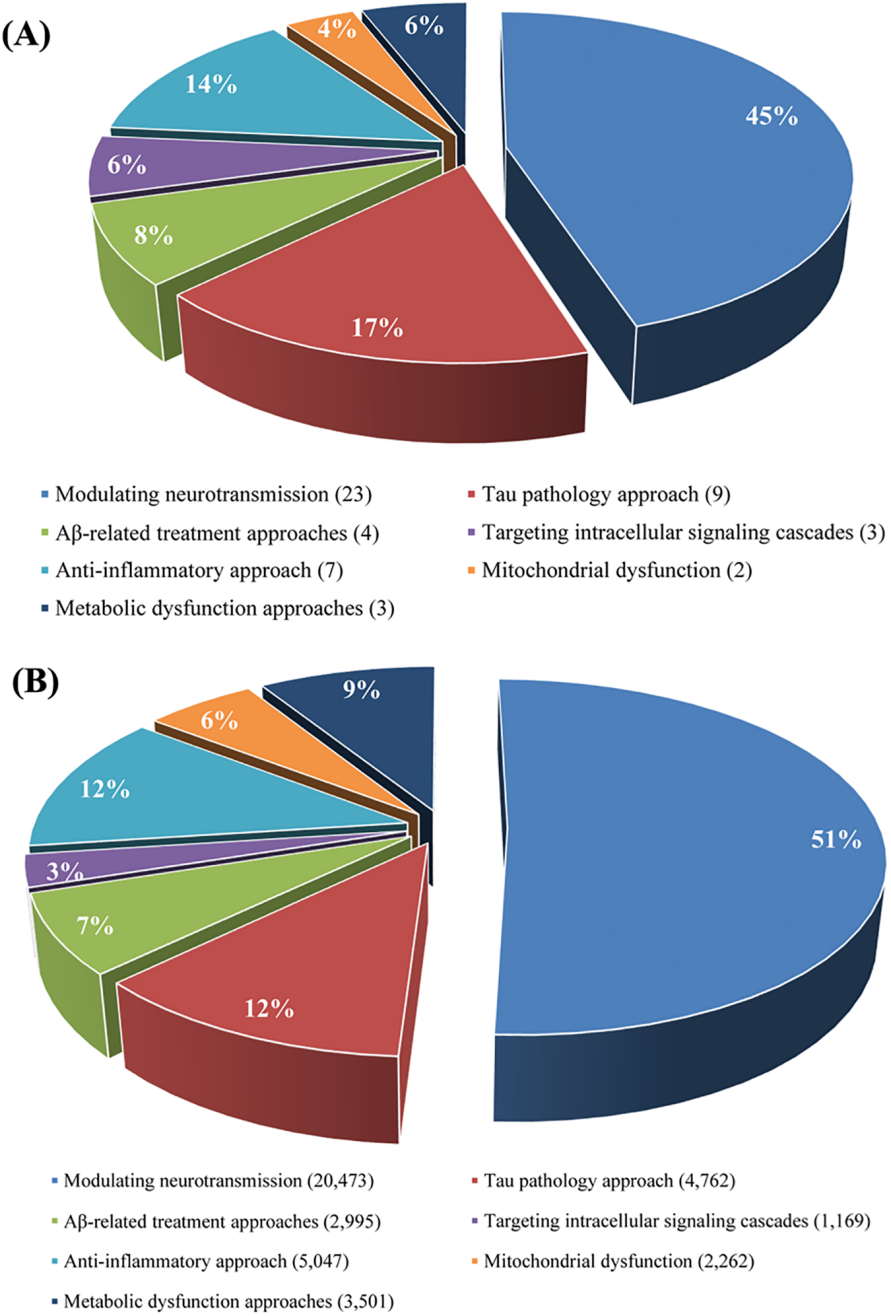
Targets (A) and active compounds (B) classification within the entire data set in AlzhCPI.

The distribution of the target and ligand space in AlzhCPI was also investigated. As presented in Fig 3A, the target space (n = 51) can be divided into seven subfamilies according to multiple mechanisms involved in the pathogenesis of AD [20], namely modulating neurotransmission (n = 23), the tau pathology approach (n = 10), Aβ-related treatment approaches (n = 4), targeting intracellular signalling cascades (n = 3), the anti-inflammatory approach (n = 7), the mitochondrial dysfunction approach (n = 2), and the metabolic dysfunction approach (n = 3). Detailed information on the target classification is given in S4 Table. The number of corresponding ligands for seven subfamilies was 20,473, 4,762, 2,995, 1,169, 5,047, 2,262 and 3,501, respectively (Fig 3B). The above analysis demonstrates that the entire data set has diverse ligand and target coverage.

The prediction quality for each sub-family were also evaluated by calculating the average MCC and AUC values in the 5-fold cross-validation (S5 Table). The high performance was obtained for each sub-family. For example, the average MCC value of NB_ECFP6 models for each sub-family ranges from 0.952 to 0.990, while their average AUC value falls in the range of 0.994 to 0.999.

### Model evaluation and comparison

The classification performance of 104 classifiers for 26 preclinical targets was evaluated, and the results are given in Tables 2 and 3. In Table 2, the statistical results for the training sets were achieved using 5-fold cross-validation. Among the 104 models, 80 classifiers out of 104 (77%) obtain an MCC value higher than 0.8, whereas 98 models out of 104 (94%) give an AUC value higher than 0.9. In general, the values of MCC range from 0.564 to 1, with an average of 0.887, whereas the values of AUC fall in the range of 0.815 to 1, with an average of 0.968. The more detailed performance of the training sets can be found in S6 Table. Furthermore, 90 out of 104 models (87%) have the values of Q higher than 0.9, with an average of 0.954. The results above indicate that the overall predictive accuracies of the mt-QSAR models are desirable.

**Table 2:** Performance of the 5-fold cross-validation for 26 targets towards Alzheimer disease using NB and RP classifiers.

Encoding Gene	ECFP6				MACCS			
	NB		RP		NB		RP	
	MCC	AUC	MCC	AUC	MCC	AUC	MCC	AUC
HTR2A	0.992	1	0.944	0.988	0.732	0.948	0.938	0.989
ADORA2A	0.989	1	0.947	0.989	0.89	0.981	0.984	0.995
CHRM2	0.984	0.999	0.877	0.976	0.779	0.963	0.928	0.978
PDE9A	0.994	0.999	0.913	0.97	0.939	0.993	0.947	0.971
GRM2	0.989	1	0.955	0.987	0.754	0.962	0.892	0.979
GRM3	1	1	0.882	0.968	0.906	0.984	0.889	0.961
MAPK8	0.991	1	0.916	0.973	0.707	0.941	0.893	0.966
MAPK9	0.98	0.996	0.852	0.961	0.763	0.945	0.822	0.939
MAPK10	0.952	0.993	0.866	0.956	0.65	0.915	0.849	0.943
MAPK14	1	1	0.905	0.935	0.916	0.98	0.795	0.897
HS90AA1	0.975	0.997	0.928	0.984	0.689	0.941	0.911	0.97
PIN1	0.978	0.999	0.914	0.964	0.978	0.998	0.812	0.922
MAPT	0.937	0.998	0.725	0.886	0.794	0.904	0.724	0.815
PTGS2	0.956	0.997	0.93	0.982	0.698	0.935	0.965	0.991
NOS2	0.976	0.999	0.886	0.968	0.702	0.929	0.887	0.97
MPO	0.956	0.996	0.914	0.963	0.781	0.956	0.918	0.953
CHUK	0.983	0.992	0.955	0.961	0.729	0.971	0.882	0.947
IKBKB	0.993	1	0.932	0.983	0.775	0.954	0.905	0.967
TNF	0.867	0.985	0.814	0.933	0.564	0.854	0.798	0.938
ALOX12	0.989	1	0.924	0.98	0.88	0.986	0.936	0.989
CTSD	0.961	0.994	0.976	0.994	0.729	0.949	0.942	0.992
PDK1	0.995	0.997	0.981	0.996	0.985	0.994	0.983	0.991
HMGCR	0.991	1	0.974	0.996	0.935	0.998	0.97	0.995
IDE	0.851	0.988	0.679	0.881	0.68	0.923	0.753	0.829
PPARG	0.981	0.998	0.955	0.991	0.745	0.947	0.934	0.988
CES1	0.956	0.999	0.934	0.972	0.676	0.913	0.89	0.969

**Table 3:** Performance of the test set validation for 25 targets towards Alzheimer disease using NB and RP classifiers.

Encoding Gene	ECFP6				MACCS			
	NB		RP		NB		RP	
	MCC	AUC	MCC	AUC	MCC	AUC	MCC	AUC
HTR2A	0.953	0.997	0.884	0.967	0.678	0.931	0.838	0.959
ADORA2A	0.653	0.949	0.681	0.911	0.553	0.868	0.26	0.714
CHRM2	0.797	0.961	0.738	0.889	0.664	0.915	0.651	0.939
PDE9A	0.96	0.994	0.836	0.954	0.643	0.982	0.771	0.855
GRM2	0.956	0.989	0.893	0.955	0.544	0.876	0.687	0.917
GRM3	0.832	0.897	0.797	0.911	0.785	0.874	0.788	0.847
MAPK8	0.927	0.991	0.801	0.928	0.651	0.903	0.746	0.898
MAPK9	0.829	0.956	0.681	0.869	0.633	0.901	0.615	0.874
MAPK10	0.787	0.937	0.695	0.879	0.541	0.852	0.594	0.84
MAPK14	0.965	0.984	0.894	0.921	0.75	0.935	0.393	0.7
HS90AA1	0.821	0.935	0.807	0.897	0.585	0.88	0.745	0.857
PIN1	0.854	0.964	0.791	0.906	0.728	0.899	0.698	0.887
MAPT	0.832	0.97	0.408	0.748	0.591	0.854	0.415	0.779
PTGS2	0.854	0.983	0.756	0.919	0.587	0.874	0.898	0.976
NOS2	0.893	0.983	0.752	0.901	0.543	0.841	0.668	0.894
MPO	0.787	0.994	0.666	0.865	0.383	0.629	0.492	0.752
CHUK	0.735	0.939	0.731	0.856	0.726	0.928	0.677	0.921
IKBKB	0.895	0.973	0.832	0.911	0.696	0.907	0.718	0.915
TNF	0.697	0.915	0.501	0.791	0.171	0.722	0.502	0.814
ALOX12	0.849	0.97	0.752	0.906	0.718	0.901	0.804	0.932
CTSD	0.885	0.974	0.92	0.95	0.647	0.913	0.867	0.941
PDK1	0.946	0.959	0.955	0.976	0.923	0.961	0.937	0.955
HMGCR	0.964	1	0.963	0.987	0.913	0.995	0.929	0.984
IDE	0.864	0.983	0.321	0.729	0.114	0.69	0.401	0.704
PPARG	0.897	0.965	0.884	0.948	0.661	0.916	0.803	0.928
CES1	0.683	0.929	0.809	0.919	0.472	0.792	0.662	0.861

To further evaluate the built mt-QSAR models, external test set validation was also performed to control the quality of the computational model. As shown in Table 3, the test sets of 104 mt-QSAR classifiers achieve an overall acceptable performance. The MCC values range from 0.114 to 0.965, with an average value of 0.724. The AUC values range from 0.629 to 1.0, with an average of 0.903. Among the 26 preclinical targets, the four models from the insulin-degrading enzyme (IDE_HUMAN) perform the worst, with average MCC and AUC values of 0.501 and 0.777, respectively. The main reason for this is that few active compounds are included in the training set (n = 60), resulting in a narrow application domain of the generated classifiers, which fails to predict the test set (n = 20). The detailed performance of the test sets is given in S7 Table.

**Fig 4:**
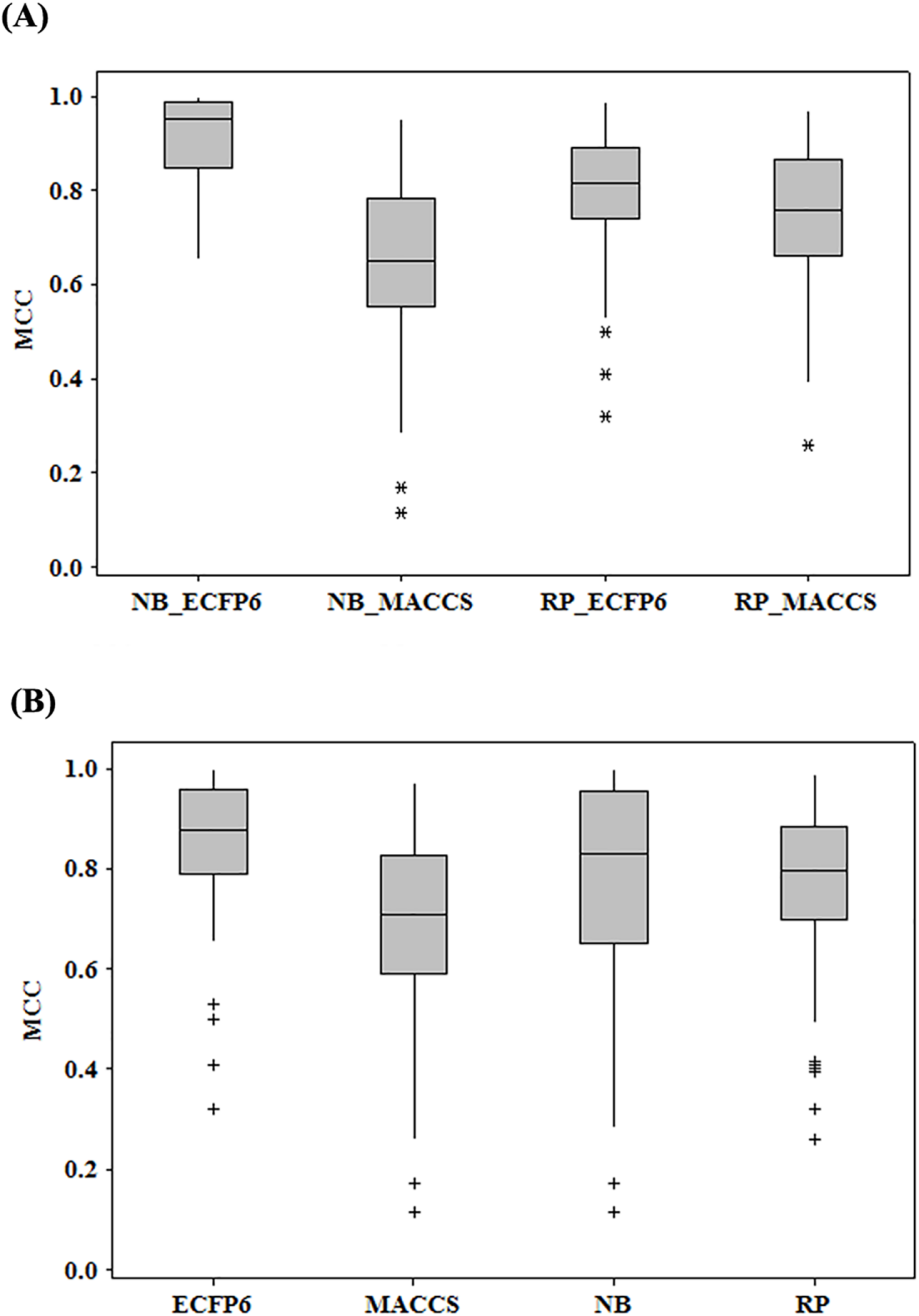
Boxplot shows the minimum, lower quartile (Q1), median (Q2), upper quartile (Q3), and maximum of Matthews correlation coefficient (MCC) on test sets based on four types of classifiers (A) and different fingerprints and algorithms (B).

The updated AlzhCPI was composed of 204 binary classifiers towards 54 important targets related to AD. To compare the performance of four types of classifiers (NB_ECFP6, NB_MACCS, RP_ECFP6 and RP_MACCS), a boxplot graph (Fig 4A) was plotted to show the minimum, lower quartile (Q1), median quartile (Q2), upper quartile (Q3), and maximum of MCC values of test sets. As shown in Fig 4A, among the four types of classifiers, the NB_ECFP6 models (Q2 = 0.953) outperform the other three, and the NB_MACCS classifiers (Q2 = 0.651) perform the worst. However, there are no obvious differences between the performance of RP_ECFP6 (Q2 = 0.816) and RP_MACCS (Q2 = 0.757). As they are based on the same fingerprint, it is interesting that the NB_ECFP6 (Q2 = 0.953) models outperform RP_ECFP6 (Q2 = 0.816), whereas the RP_MACCS (Q2 = 0.757) models outperform than NB_MACCS (Q2 = 0.651). This indicates that the performance of the models derived from the different algorithms depends on which fingerprint is used.

Similarly, Fig 4B depicts the distributions of the MCC values based on the different fingerprints and algorithms. The boxplot result indicates that the classifiers (Q2 = 0.879) derived from the ECFP6 fingerprint outperform those (Q2 = 0.708) derived from the MACCS fingerprint. In addition, there is a significant difference in the performance of the NB (Q2 = 0.832) and RP (Q2 = 0.798) models. Thus, the same conclusion can be drawn that both algorithms have their respective advantages. More detailed data for the boxplot can be found in S8 Table.

As discussed above, it is necessary to integrate the results of the four single classifiers to predict CPIs. In fact, the advantage of integrated model to identify CPI has been displayed in our previous study, resulting in several highly active MTDLs against AD. In this study, the same integrated criteria is adopted. We defined CPI as a potential interaction if the molecule was forecast to be active by at least two out of the four single classifiers within one target [17].

### Implementation of AlzhCPI

In the present study, the multi-target quantitative structure-activity relationship (mt-QSAR) method using naive Bayesian (NB) and recursive partitioning (RP) algorithms was conducted. A web server, namely AlzhCPI, was designed using HTML and CSS technology to provide all the results of our models. In this web server, users can find important fragments for multi-targets against AD given by the naive Bayesian classifier, the case study of the prediction of polypharmacology for known AD drugs, and the detailed 204 binary classifiers towards 54 important targets related to AD. In addition, the users can also download the XML files of 204 models and import them to the PipelinePilot/Discovery Studio software to predict the activities of a given molecule. We anticipate that this server will facilitate the target identification and virtual screening of active compounds for the treatment of AD.

### Case study based on AlzhCPI: Systematic analysis of the multiple bioactivities of shichangpu through a network pharmacology approach

AD is caused by multiple genes or their products. Single-target therapy has been found ineffective due to insufficient understanding of the complex disease. Traditional Chinese medicine (TCM), which treats disease based on the concept of “multiple components and multiple targets”, has accumulated rich theories and a great deal of valuable experience in the prevention and treatment of AD [42]. Shichangpu is the most frequently used herbal medicine among anti-AD TCM prescriptions [43–45]. Thus, it is urgently needed to systematically analyse the mechanisms of action of shichangpu from a holistic perspective.

**Fig 5:**
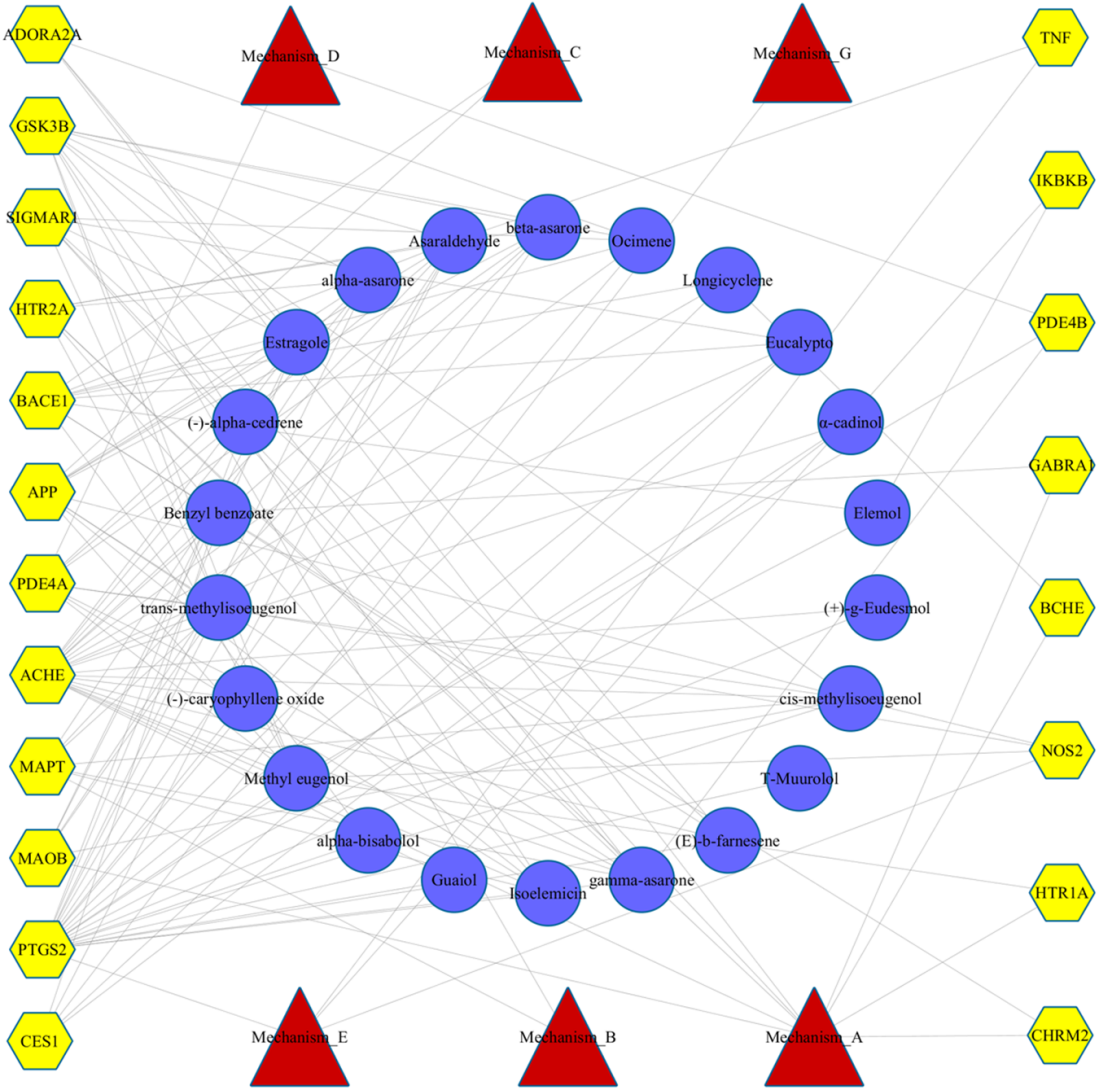
The compound–target–mechanism network of shichangpu based on AlzhCPI. Ellipse, hexagon and triangle represent drug nodes, protein nodes and mechanism nodes, respectively.

Based on AlzhCPI, the potential targets of 22 key compounds of shichangpu against AD were identified, and the associations between the molecules and target proteins are listed in S9 Table. The predicted results were also integrated to construct the compound–target–mechanism network. As shown in Fig 5, shichangpu can target 20 targets from a holistic perspective, which includes six mechanisms involved in the pathogenesis of AD. This means that shichangpu can treat AD through modulating neurotransmission, the tau pathology approach, the metabolic dysfunction approach, Aβ-related treatment, the anti-inflammatory approach and intracellular signalling cascade approach.

The degree analysis revealed that the target could interact with multiple molecules (5.75 compounds per target on average), and one compound could also target several proteins related to AD (5.23 targets per compound on average). There were 13 compounds out of 22 that could target at least 5 proteins, which may imply that these compounds are the main pharmacological active ingredients. Among the 13 compounds, both methyl eugenol and asaraldehyde were predicted to ne active against 10 targets. In addition, 10 targets out of 20 could simultaneously interact with at least 5 compounds. Among the 10 proteins, ACHE and PTGS2 achieved the highest degree (n = 21 and 18, respectively) of linking to molecular nodes, indicating that they would have key pharmacological functions in shichangpu.

## Conclusion

In this paper, based on the naive Bayesian (NB) and recursive partitioning (RP) algorithms, a model library first built in a previous study was updated by constructing 104 binary classifiers against 26 preclinical AD targets using the mt-QSAR method. The internal 5-fold cross-validation and external test set validation confirmed the prediction reliability of the models.

In addition, a web server entitled AlzhCPI was implemented to provide comprehensive information on the approximately 204 binary classifiers and is available free to the scientific community. A case for AlzhCPI was illustrated to systematically analyse the multiple bioactivities of shichangpu through a network pharmacology approach. The results showed that shichangpu could target 20 targets related to AD, which were involved in multiple mechanisms, supporting the TCM theme of “multiple components and multiple targets”.

AlzhCPI has potential applications in network pharmacology, drug repositioning, and virtual screening for MTDLs towards AD. The methodology and tools here may provide guidance for constructing similar platforms for other complex diseases.

## Supporting information

S1 Table Detailed information on the 51 targets. (XLSX)

S2 Table The generation of decoy compounds. (XLSX)

S3 Table The SMILES structures of 22 key compounds in shichangpu. (XLSX)

S4 Table The detailed information on the target classification for 51 targets. (XLSX)

S5 Table The prediction quality for each sub-family. (XLSX)

S6 Table The performance of the 5-fold cross-validation for 26 targets towards Alzheimer’s disease using NB and RP classifiers. (XLSX)

S7 Table The performance of the test set validation for 26 targets towards Alzheimer’s disease using NB and RP classifiers. (XLSX)

S8 Table The detailed parameter information from the boxplot of the test sets. (XLSX)

S9 Table The associations between molecules and targets predicted by AlzhCPI for shichangpu. (XLSX)
